# Supplementary material for: The functional significance of the RPA- and PCNA-dependent recruitment of Pif1 to DNA
Source: EMBO Rep. 2024 Mar 13;25(4):10. doi: 10.1038/s44319-024-00114-9 (PMC11014909; doi:10.1038/s44319-024-00114-9)
Supplement: Supplementary file 1 — Table EV1 [file 44319_2024_114_MOESM1_ESM.docx]

Table EV1. *Saccharomyces cerevisiae* A364a strains used in the study

| **Strain number** | **Genotype** | **Origin/References** |
| --- | --- | --- |
| NK1 | *MATa ura3-52 trp1-289 leu2-3,112 bar1::LEU2* | Makovets *et al.*, 2004 |
| NK828 | *MATa bar1::LEU2 trp1-289 ura3-5 leu2-3,112 pif1-m2* | Makovets & Blackburn, 2009 |
| NK1324 | *MATa-inc trp1-289 ura3::NAT leu2::LEU2-P_GAL1_-HO HEM13::HOsite-URA3 pif1-m2* |  |
| NK1325 | *MATa-inc trp1-289 ura3::NAT leu2::LEU2- P_GAL1_-HO HEM13::HOsite-URA3 pif1-m2-TRP1-pif1-m1* |  |
| NK1335 | *MATa-inc trp1-289 ura3::NAT leu2::LEU2- P_GAL1_-HO HEM13::HOsite-URA3 pif1-m2-TRP1-pif1-m1-4myc* |  |
| NK3728 | *MATa-inc trp1-289 ura3::NAT leu2::LEU2- P_GAL1_-HO*  *MNT2::kan::HOsite-URA3-STAR-TEL HIS7::kan pif1-m2* | Vasianovich *et al.*, 2014 |
| NK3729 |  |  |
| NK7380 | *MATa-inc trp1-289 ura3::NAT leu2::LEU2- P_GAL1_-HO*  *MNT2::kan::HOsite-URA3-STAR-TEL HIS7::kan pif1-m2-TRP1-pif1-m1-pip* | NK3728 *pif1-m2*::pYT540/BglII  (here and elsewhere *pip* = R3E, Buzovetsky *et al.*, 2017) |
| NK7381 |  |  |
| NK7382 |  | NK3729 *pif1-m2*::pYT540/BglII |
| NK7448 | *MATa-inc trp1-289 ura3::NAT leu2::LEU2- P_GAL1_-HO*  *MNT2::kan::HOsite-URA3-STAR-TEL HIS7::kan pif1-m2-TRP1-pif1-m1* | NK3728 *pif1-m2*::pYT147/BglII |
| NK7449 |  | NK3729 *pif1-m2*::pYT147/BglII |
| NK7468 | *MATa-inc trp1-289 ura3::NAT leu2::LEU2- P_GAL1_-HO HEM13::HOsite-URA3 KAN pif1-m2-TRP1-pif1-m1-pip-4myc* | NK1324 *pif1-m2*::pYT541/BglII |
| NK7469 |  |  |
| NK7470 |  |  |
| NK7511 | *MATa bar1::LEU2 trp1-289 ura3-5 leu2-3,112 rrm3::KAN pif1-m2* | NK828 *rrm3::KAN* |
| NK7512 |  |  |
| NK7513 |  |  |
| NK7614 | *MATa bar1::LEU2 trp1-289 ura3-5 leu2-3,112 rrm3::KAN pif1-m2-TRP1-pif1-m1* | NK7511 *pif1-m2*::pYT147/BglII |
| NK7615 |  |  |
| NK7616 |  | NK7512 *pif1-m2*::pYT147/BglII |
| NK7617 | *MATa bar1::LEU2 trp1-289 ura3-5 leu2-3,112* *rrm3::KAN pif1-m2-TRP1-pif1-m1-pip* | NK7511 *pif1-m2*::pYT540/BglII |
| NK7618 |  |  |
| NK7619 |  | NK7512 *pif1-m2*::pYT147/BglII |
| NK7626 | *MATa bar1::LEU2 trp1-289 ura3-5 leu2-3,112* *rrm3::KAN pif1-m2-TRP1-pif1-m1-rbm* | NK7511 *pif1-m2*::pYT636/BglII |
| NK7627 |  |  |
| NK7628 |  | NK7512 *pif1-m2*::pYT636/BglII |
| NK7638 | *MATa-inc trp1-289 ura3::NAT leu2::LEU2- P_GAL1_-HO*  *MNT2::kan::HOsite-URA3-STAR-TEL HIS7::kan pif1-m2-TRP1-pif1-m1-rbm* | NK3728 *pif1-m2*::pYT636/BglII |
| NK7639 |  |  |
| NK7640 |  | NK3729 *pif1-m2*::pYT636/BglII |
| NK7650 | *MATa-inc trp1-289 ura3::NAT leu2::LEU2- P_GAL1_-HO HEM13::HOsite-URA3 KAN pif1-m2-TRP1-pif1-m1-rbm-4myc* | NK1324 *pif1-m2*::pYT623/BglII |
| NK8120 | *MATa trp1-289 leu2-3,112 bar1::LEU2 ura3-52:: P_GAL1_-pif1-m1-4myc* | NK1 *ura3*::pYT730/StuI |
| NK8121 |  |  |
| NK9925 | *MATa trp1-289 leu2-3,112 bar1::LEU2 ura3-52:: P_GAL1_-pif1-m1-rbm-4myc* | NK1 *ura3*::pYT739/StuI |
| NK9926 |  |  |
| NK10216 | *MATa-inc trp1-289 ura3::NAT leu2::LEU2- P_GAL1_-HO*  *MNT2::kan::HOsite-URA3-STAR-TEL HIS7::kan pif1-m2-TRP1-pif1-m1-rbm-pip* | NK3728 *pif1-m2*::pYT1176/BglII |
| NK10217 |  |  |
| NK10218 |  | NK3729 *pif1-m2*::pYT1176/BglII |
| NK10219 |  |  |
| NK10620 | *MATa-inc trp1-289 ura3::NAT leu2::LEU2- P_GAL1_-HO HEM13::HOsite-URA3 KAN pif1-m2-TRP1-pif1-m1-rbm-4myc* | NK1324 *pif1-m2*::pYT623/BglII |
| NK10621 |  |  |
| NK10623 | *MATa-inc trp1-289 ura3::NAT leu2::LEU2- P_GAL1_-HO HEM13::HOsite-URA3 KAN pif1-m2-TRP1-pif1-m1-rbm-4myc* | NK1324 *pif1-m2*::pYT1177/BglII |
| NK10624 |  |  |
| NK10625 |  |  |
| NK10626 | *MATa bar1::LEU2 trp1-289 ura3-5 leu2-3,112* *rrm3::KAN pif1-m2-TRP1-pif1-m1-rbm-pip* | NK7511 *pif1-m2*::pYT1176/BglII |
| NK10627 |  |  |
| NK10628 |  |  |
| NK10665 | *MATa trp1-289 leu2-3,112 bar1::LEU2 ura3-52:: P_GAL1_-pif1-m1-pip-4myc* | NK1 *ura3*::pYT744/StuI |
| NK10666 |  |  |
| NK10717 | *MATa-inc trp1-289 ura3::NAT leu2::LEU2- P_GAL1_-HO MNT2::kan::HOsite-URA3-STAR-TEL HIS7::kan pif1-m2-TRP1- P_GAL1_-pif1-m1-rbm-4myc* | NK3728 *pif1-m2*::pYT1269/BglII |
| NK10718 |  | NK3729 *pif1-m2*::pYT1269/BglII |
| NK10719 |  |  |
| NK10720 | *MATa-inc trp1-289 ura3::NAT leu2::LEU2- P_GAL1_-HO MNT2::kan::HOsite-URA3-STAR-TEL HIS7::kan pif1-m2-TRP1- P_GAL1_-pif1-m1-rbm-pip-4myc* | NK3728 *pif1-m2*::pYT1270/BglII |
| NK10721 |  | NK3729 *pif1-m2*::pYT1270/BglII |
| NK10722 |  |  |
| NK10804 | *MATa trp1-289 leu2-3,112 bar1::LEU2 ura3-52:: P_GAL1_-pif1-m1-K264A-4myc* | NK1 *trp1*::pYT1264/EcoRV |
| NK10805 |  |  |
| NK10893 | *MATa bar1::LEU2 trp1-289 ura3-5 leu2-3,112 rrm3::KAN pif1-m2-TRP1-pif1-m1 [pRS426]* | NK7614 [pRS426] |
| NK10895 |  | NK7616 [pRS426] |
| NK10896 | *MATa bar1::LEU2 trp1-289 ura3-5 leu2-3,112 rrm3::KAN pif1-m2 [pRS426]* | NK7511 [pRS426] |
| NK10898 |  | NK7512 [pRS426] |
| NK10899 | *MATa bar1::LEU2 trp1-289 ura3-5 leu2-3,112* *rrm3::KAN pif1-m2-TRP1-pif1-m1-rbm [pRS426]* | NK7626 [pRS426] |
| NK10901 |  | NK7628 [pRS426] |
| NK10902 | *MATa bar1::LEU2 trp1-289 ura3-5 leu2-3,112* *rrm3::KAN pif1-m2-TRP1-pif1-m1-pip [pRS426]* | NK7617 [pRS426] |
| NK10904 |  | NK7619 [pRS426] |
| NK10905 | *MATa bar1::LEU2 trp1-289 ura3-5 leu2-3,112* *rrm3::KAN pif1-m2-TRP1-pif1-m1-rbm-pip [pRS426]* | NK10626 [pRS426] |
| NK10907 |  | NK10628 [pRS426] |
| NK10934 | *MATa bar1::LEU2 trp1-289 ura3-5 leu2-3,112* | NK1 [pRS426] |
| NK10935 |  | NK1 [pRS426] |
