# Supplementary material for: The functional significance of the RPA- and PCNA-dependent recruitment of Pif1 to DNA
Source: EMBO Rep. 2024 Mar 13;25(4):10. doi: 10.1038/s44319-024-00114-9 (PMC11014909; doi:10.1038/s44319-024-00114-9)
Supplement: Supplementary file 2 — Table EV2 [file 44319_2024_114_MOESM2_ESM.docx]

Table EV2. Plasmids used in the study

| **Plasmid** | **Backbone** | **Insert** | **Purpose** |
| --- | --- | --- | --- |
| pYT147 | pRS404 | *P_PIF1_-pif1-m1-T_PIF1_* | To introduce *pif1* mutations into the yeast genome  (here and elsewhere *pip* = R3E, Buzovetsky *et al.*, 2017) |
| pYT540 | pRS404 | *P_PIF1_-pif1-m1-pip-T_PIF1_* |  |
| pYT541 | pRS404 | *P_PIF1_-pif1-m1-pip-4myc-T_PIF1_* |  |
| pYT623 | pRS404 | *P_PIF1_-pif1-m1-rbm-T_PIF1_* |  |
| pYT636 | pRS404 | *P_PIF1_-pif1-m1-rbm-4myc-T_PIF1_* |  |
| pYT730 | pRS406 | *P_GAL1_-pif1-m1-4myc-T_PIF1_* | Plasmid for Pif1 overproduction in yeast |
| pYT739 | pRS406 | *P_GAL1_-pif1-m1-rbm-4myc-T_PIF1_* | Plasmid for Pif1-rbm overproduction in yeast |
| pYT744 | pRS406 | *P_GAL1_-pif1-m1-pip-4myc-T_PIF1_* | Plasmid for Pif1-pip overproduction in yeast |
| pYT928 | pGEX-4T-2 | *GST-6Gly-pif1N(40-250)* | To produce GST/GST-Pif1N fusions in *E.coli* |
| pYT1094 | pGEX-4T-2 | *GST-2Gly* |  |
| pYT1117 | pGEX-4T-2 | *GST-6Gly-pif1N(40-250)-rbm^-^* |  |
| pYT1120 | pETM-33 | *rfa1N(1-122)-4myc* | To produce Rfa1N-4myc in *E. coli* |
| pYT1125 | pGEX-4T-2 | *GST-6Gly-pif1C(750-859)* | To produce GST-Pif1C fusion in *E.coli* |
| pYT1176 | pRS404 | *P_PIF1_-pif1-m1-rbm-pip-T_PIF1_* | To introduce *PIF1* mutations into the yeast genome |
| pYT1177 | pRS404 | *P_PIF1_-pif1-m1-rbm-pip-4myc-T_PIF1_* |  |
| pYT1178 | pGEX-4T-2 | *GST-6Gly-Gly-Ser-pif1N(56-77)* | To produce GST-Pif1 fusions in *E.coli* |
| pYT1179 | pGEX-4T-2 | *GST-6Gly-Gly-Ser-pif1N(56-77)-rbm^-^* |  |
| pYT1264 | pRS404 | *P_GAL1_-pif1-m1-hd(K264A)-4myc-T_PIF1_* | Plasmids for *pif1-hd* mutant allele overexpression in yeast |
| pYT1270 | pRS404 | *P_GAL1_-pif1-m1-rbm-pip-4myc-T_PIF1_* |  |
