# Supplementary material for: The functional significance of the RPA- and PCNA-dependent recruitment of Pif1 to DNA
Source: EMBO Rep. 2024 Mar 13;25(4):10. doi: 10.1038/s44319-024-00114-9 (PMC11014909; doi:10.1038/s44319-024-00114-9)
Supplement: Supplementary file 3 — Table EV3 [file 44319_2024_114_MOESM3_ESM.docx]

Table EV3. Oligonucleotides used in the study

| **Oligonucleotide number** | **Sequence (5’ to 3’)** | **Purpose** |
| --- | --- | --- |
| OSM60 | CCCACACTTTTCACATCTACCTCTACTCTCGCTGTCACTCCTTACCCGGC | To amplify the DNA fragments for KL1 probe Makovets *et al.*, 2004 |
| OSM106 | CCCCGAATTCCGGCATTCCTGTCGATGCTGATAGGG |  |
| OSM523 | TCATGTACGTCTCCTCCAAGCCC | qPCR at Y’-telomeres |
| OSM524 | GCAGTAGCGAGAGACAAGTGGGAAA |  |
| OSM532 | CCCCGGTACCGGTTTGCCTACAAGATAAAAGTAAATT | To amplify the DNA fragments for *MNT2* probe Makovets & Blackburn, 2009 |
| OSM533 | CCCCGAATTCGAATTACGTGAATATTATGCATCACC |  |
| OSM559 | ACAAGGCATATAGGCGCAGCTTCA | qPCR at DSB site (*HEM13*) |
| OSM560 | ACCTGGCGATAGCCTTCTGGTAAA |  |
| OSM1006 | TGACTGGTACTACCGTAACGGTTC | qPCR at *ARO1* locus |
| OSM1007 | GAATACCATCTGGTAATTCTGTAGTTTTGAC |  |
| OSM2161 | TGTGGATATCTTGACTGATTTTTCC | To make *URA3* probe |
| OSM2162 | ATACATGCATTTACTTATAATACAG |  |
| OSM2881 | AGAGCAGCGCGACGTCAAGAGCACAGTAGGAAGAGATTTCAGTTG | To mutate the PIP motif in Pif1 (R3E, Buzovetsky *et al.*, 2017) |
| OSM2882 | CTCTTGACGTCGCGCTGCTCTACCATTATTAGATTGTGTGGTCGC |  |
| OSM3280 | CCCCCGGCCGCGTCTTCTTTTGAAAGTATGGAA | To mutate the RBM motif in Pif1 |
| OSM3281 | CCCCCGGCCGCTGCTTCTGATTCGGATGATTGGGA |  |
| OSM3299 | TGCACTCACACCATTCACACT | To amplify the DNA fragments for a probe in the *tA(AGC)F* locus |
| OSM3300 | ATTGGCCCAAAAGGGATCAT |  |
| OSM3432 | CCCCGGATCCGGTGGAGGTGGAGGTGGAATGAGTAGTCGTGGTTTCAGG | To make *GST-6Gly-pif1N(40-250)* fusion for expression in *E. coli* |
| OSM3570 | CCCCGTCGACTTAATTTTCTGCCAACTTGATGATACTTTC |  |
| OSM4159 | CCCCTCTAGAAATAATTTTGTTTAACTTTAAGAAGGAGATATACCATGAGCAGTGTTCAACTTTCG | To make the plasmid for *rfa1N(1-122)-4myc* expression in *E. coli* |
| OSM4160 | CCCCGGATCCGACCACCGCCTCCACCGTTATCCAAAAAAGTACTAGTTTG |  |
| OSM4165 | CCCCGTCGACTTATTCTTCCCAATCATCCGAATC | To make *GST-6Gly-Ser-Gly-pif1N(56-77)* fusion for expression in *E. coli* |
| OSM4279 | CCCCGGATCCAAACACCCGAGCATTCTGAGCAAAGAG |  |
