# Supplementary figures and images for: The functional significance of the RPA- and PCNA-dependent recruitment of Pif1 to DNA
Source: EMBO Rep. 2024 Mar 13;25(4):10. doi: 10.1038/s44319-024-00114-9 (PMC11014909; doi:10.1038/s44319-024-00114-9)

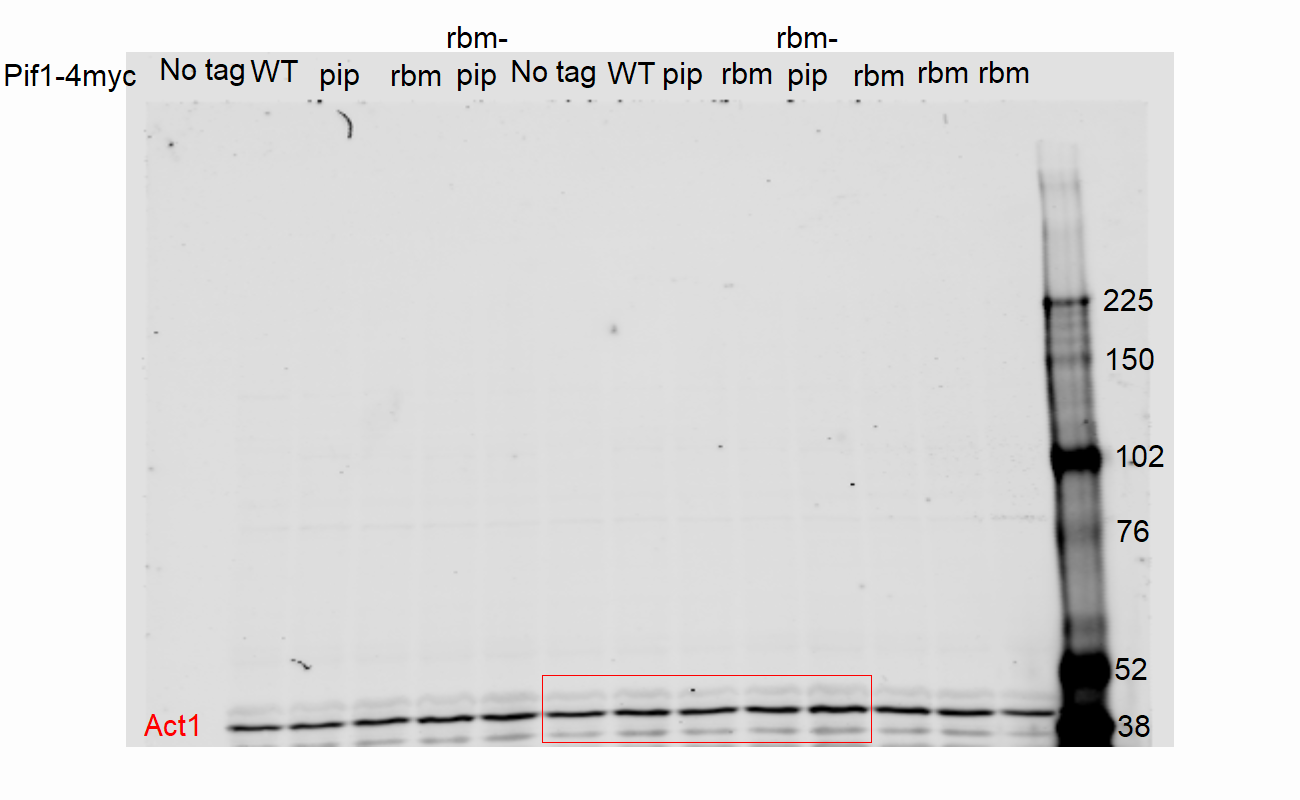

Supplement: Supplementary file 4 — Source Data for EV Figures [file 44319_2024_114_MOESM4_ESM.zip › Figure EV1/Act1 blot labelled.tif]

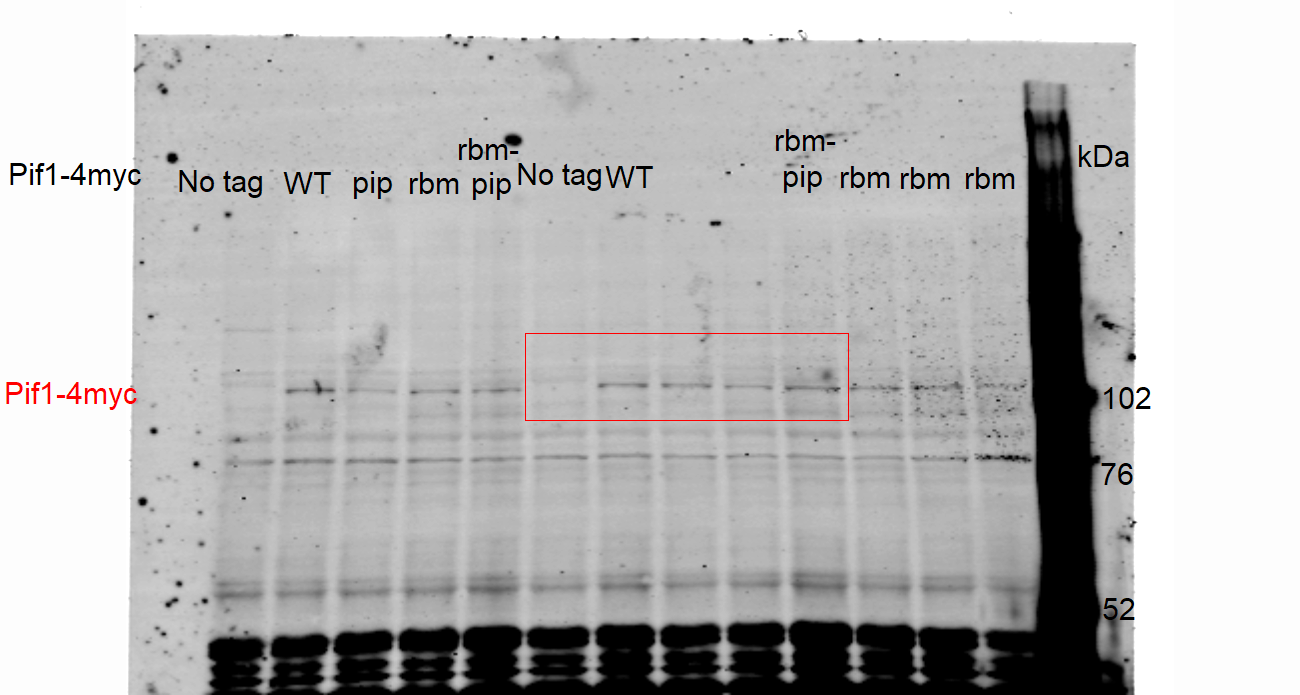

Supplement: Supplementary file 4 — Source Data for EV Figures [file 44319_2024_114_MOESM4_ESM.zip › Figure EV1/myc blot labelled.tif]

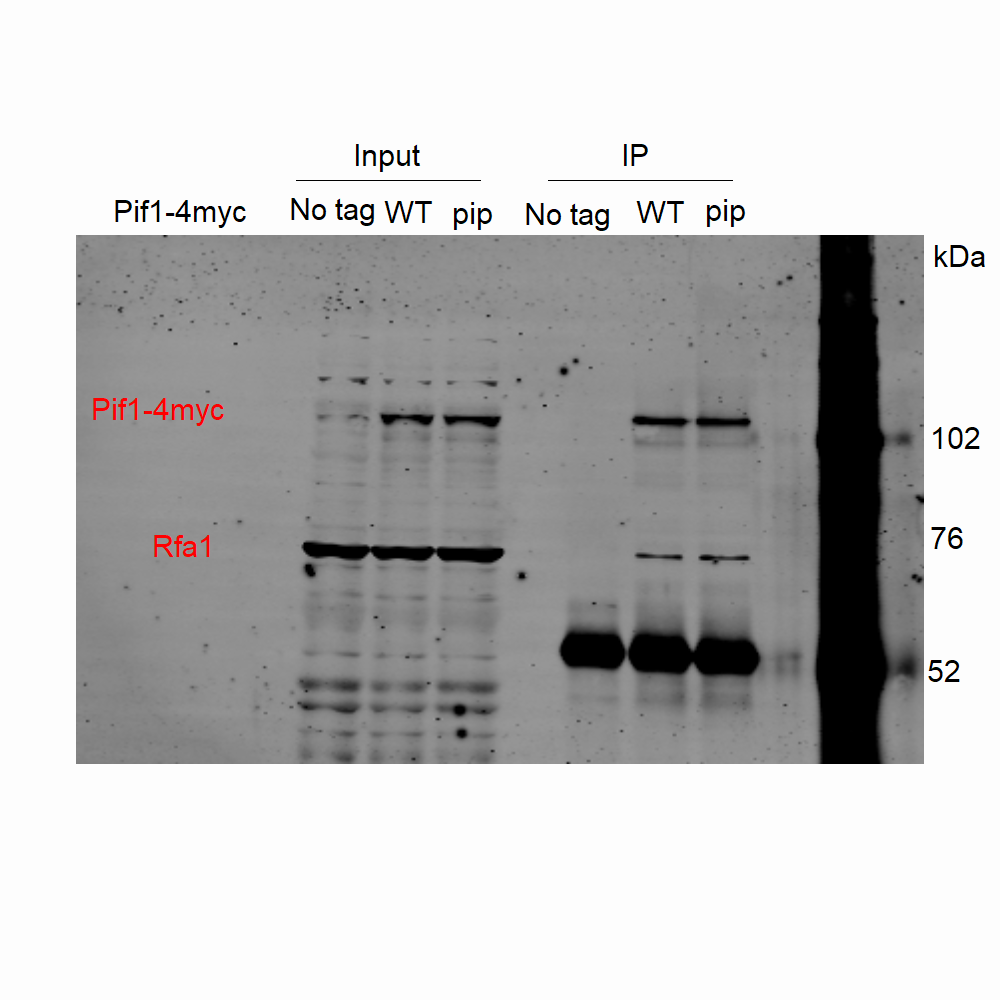

Supplement: Supplementary file 4 — Source Data for EV Figures [file 44319_2024_114_MOESM4_ESM.zip › FIgure EV2/Pif1 and Rfa1 blot labelled.tif]

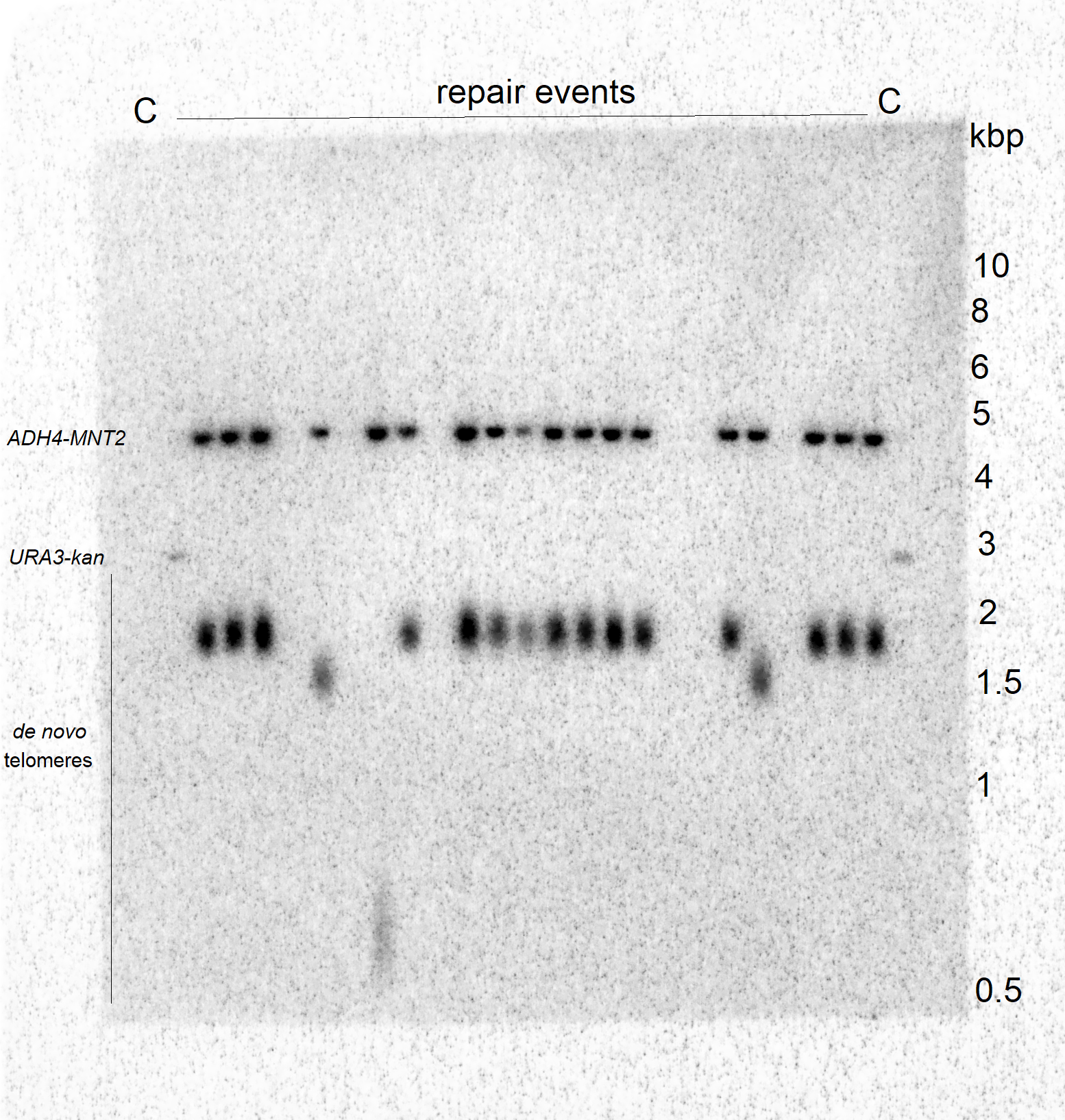

Supplement: Supplementary file 4 — Source Data for EV Figures [file 44319_2024_114_MOESM4_ESM.zip › Figure EV3/MNT2 blot labelled.tif]

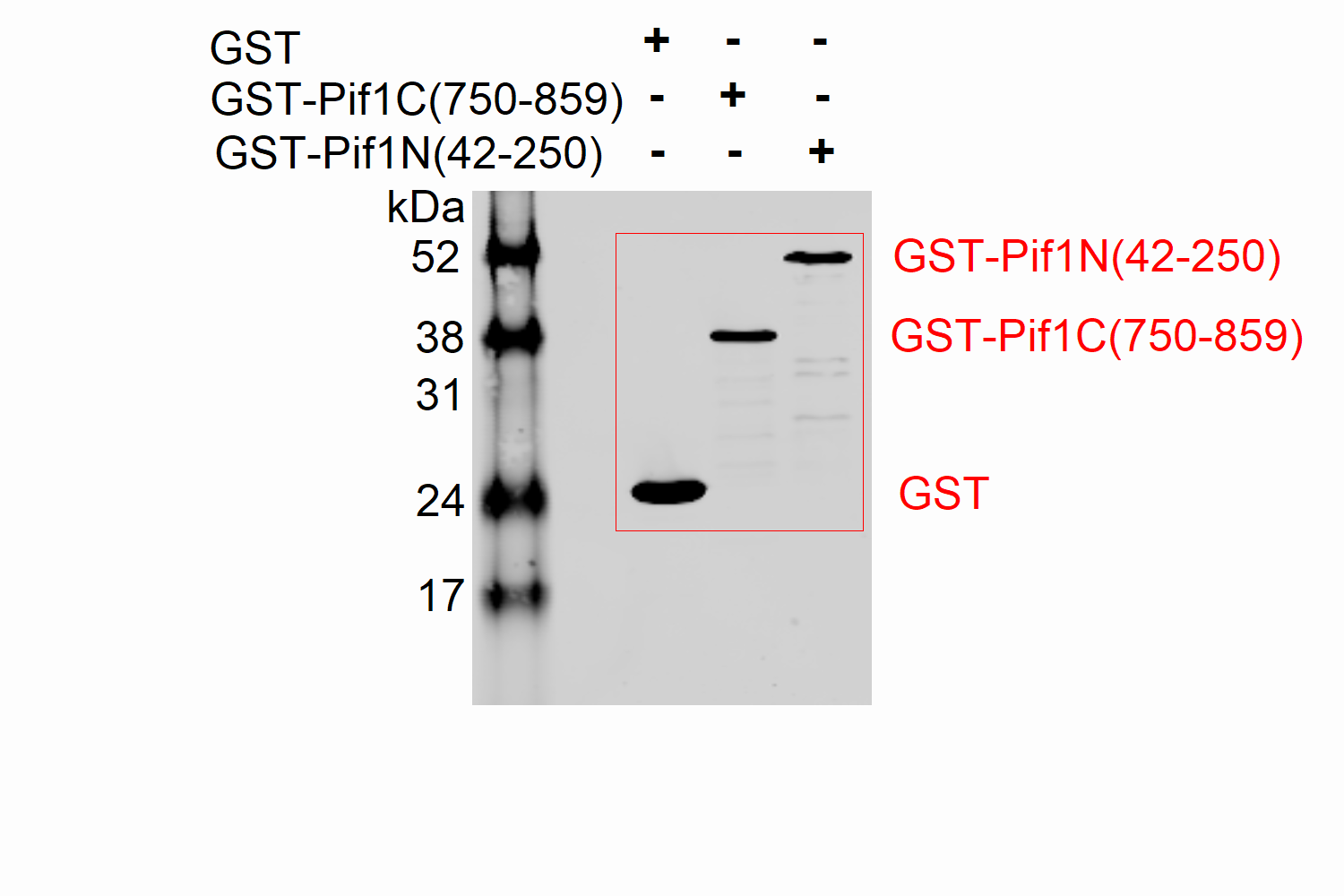

Supplement: Supplementary file 5 — Source Data Fig. 1 [file 44319_2024_114_MOESM5_ESM.zip › Panels A and B/western GST.tif]

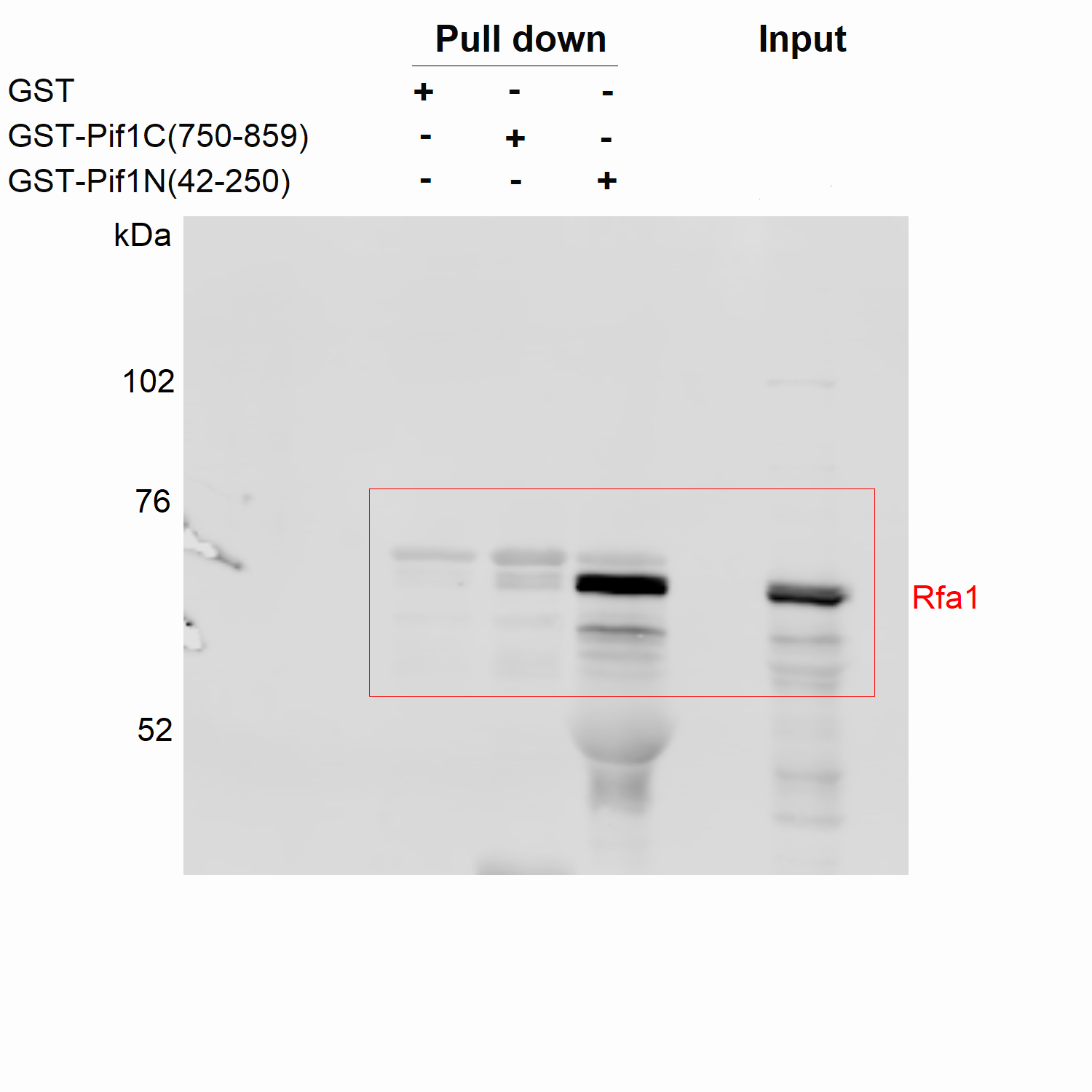

Supplement: Supplementary file 5 — Source Data Fig. 1 [file 44319_2024_114_MOESM5_ESM.zip › Panels A and B/western Rfa1.tif]

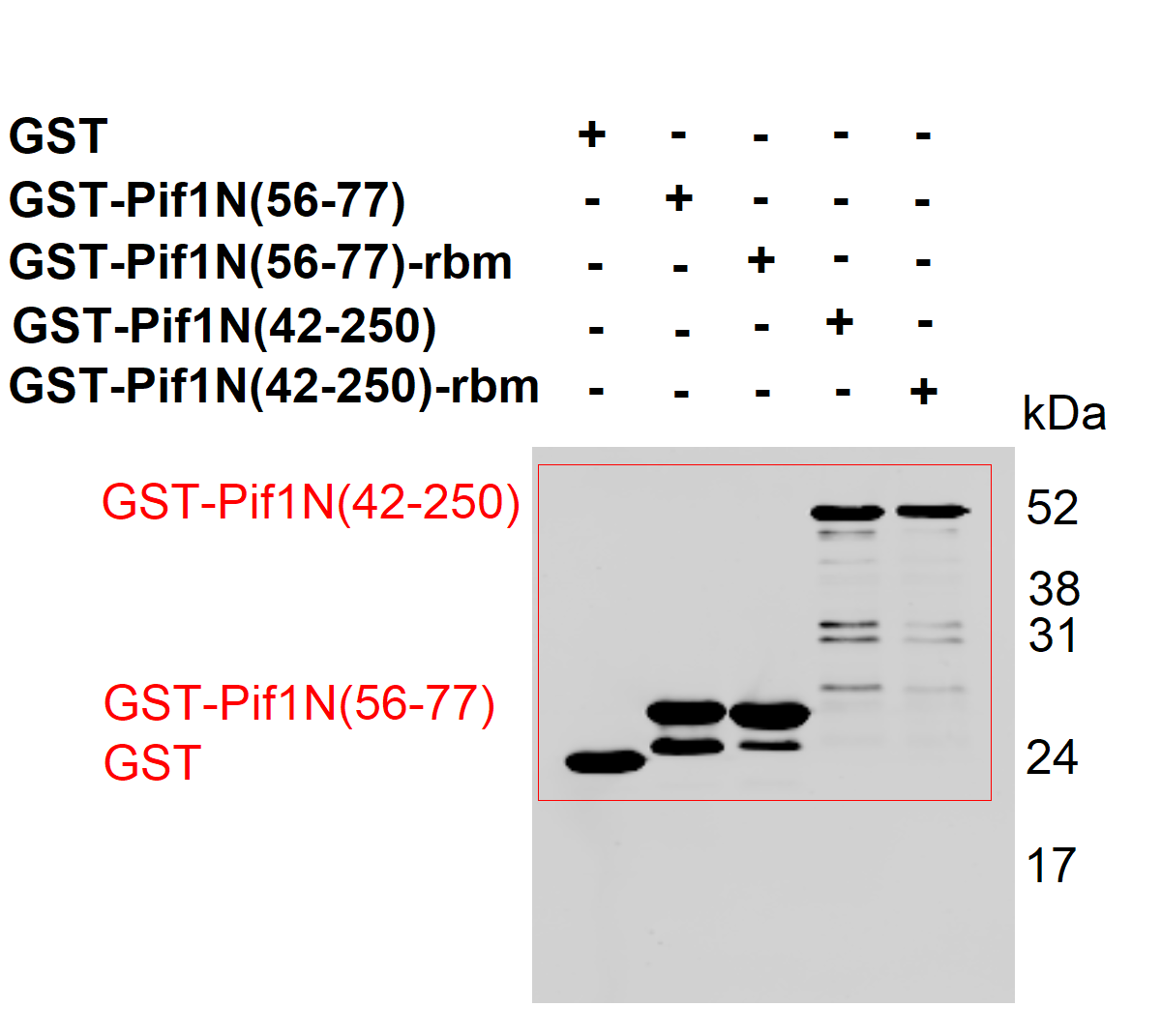

Supplement: Supplementary file 5 — Source Data Fig. 1 [file 44319_2024_114_MOESM5_ESM.zip › Panels D and E/western GST.tif]

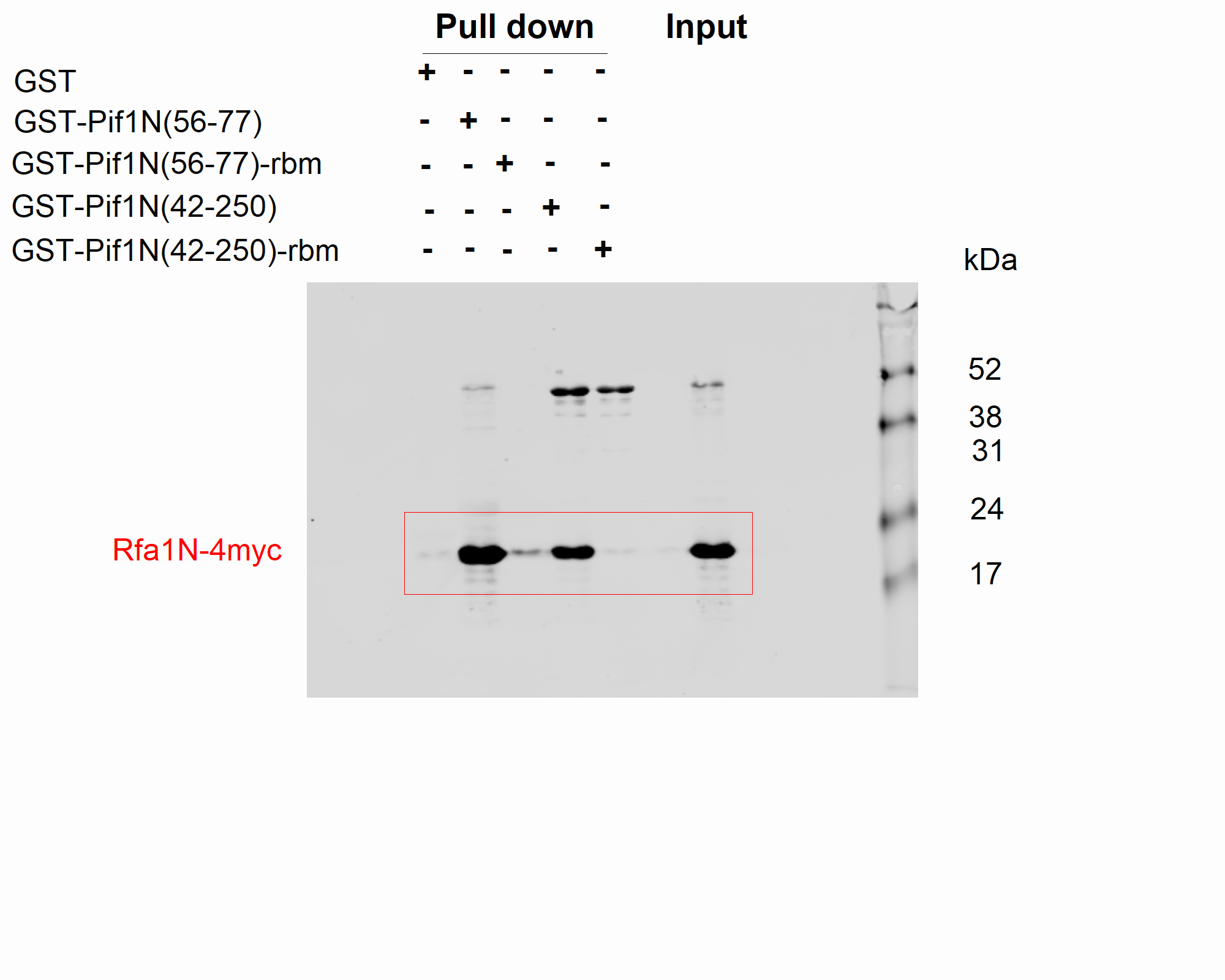

Supplement: Supplementary file 5 — Source Data Fig. 1 [file 44319_2024_114_MOESM5_ESM.zip › Panels D and E/western myc.tif]

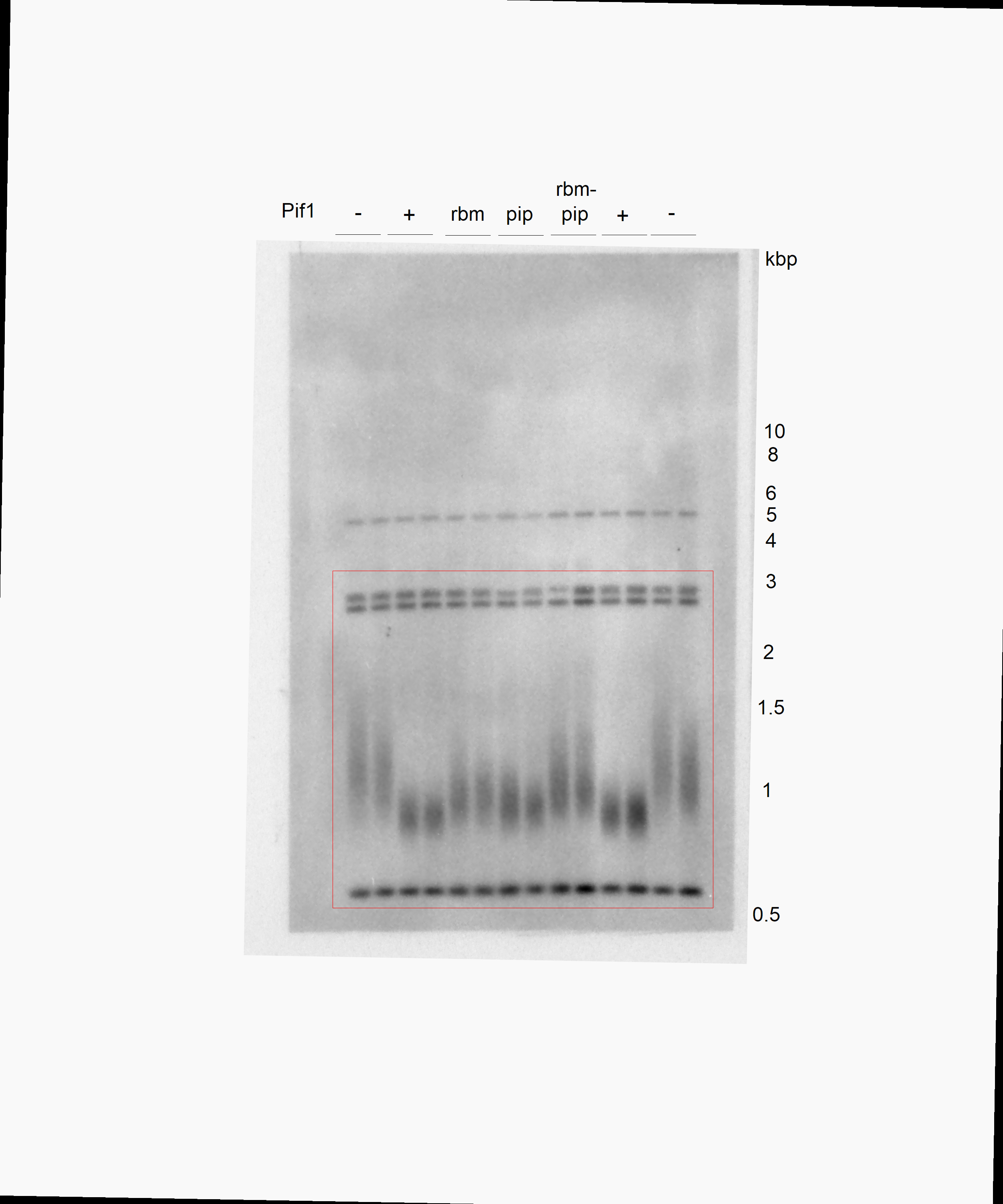

Supplement: Supplementary file 6 — Source Data Fig. 2 [file 44319_2024_114_MOESM6_ESM.zip › Panel F/southern teloblot.tif]

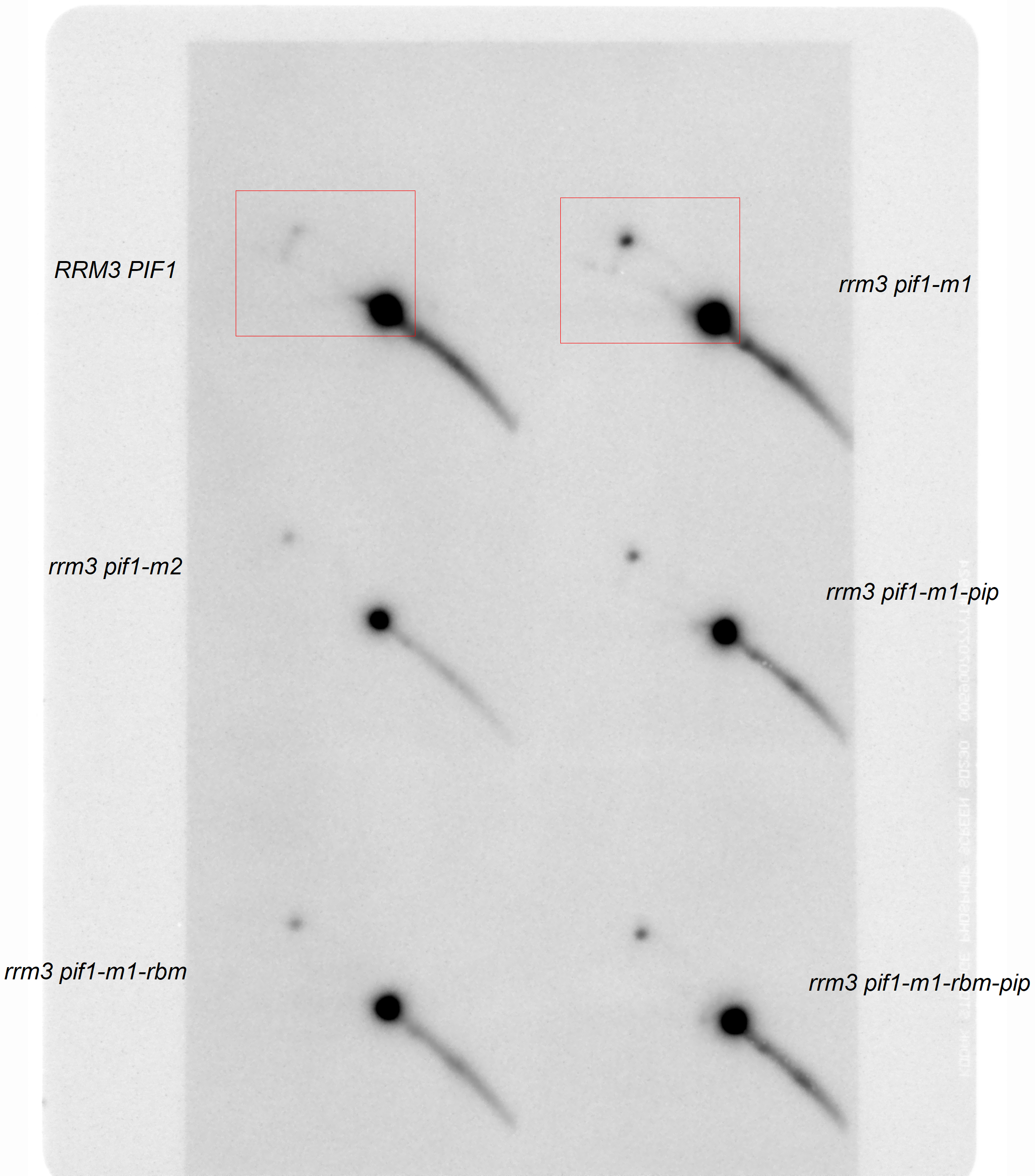

Supplement: Supplementary file 7 — Source Data Fig. 3 [file 44319_2024_114_MOESM7_ESM.zip › Panel B/tRNA southern part 1.tif]

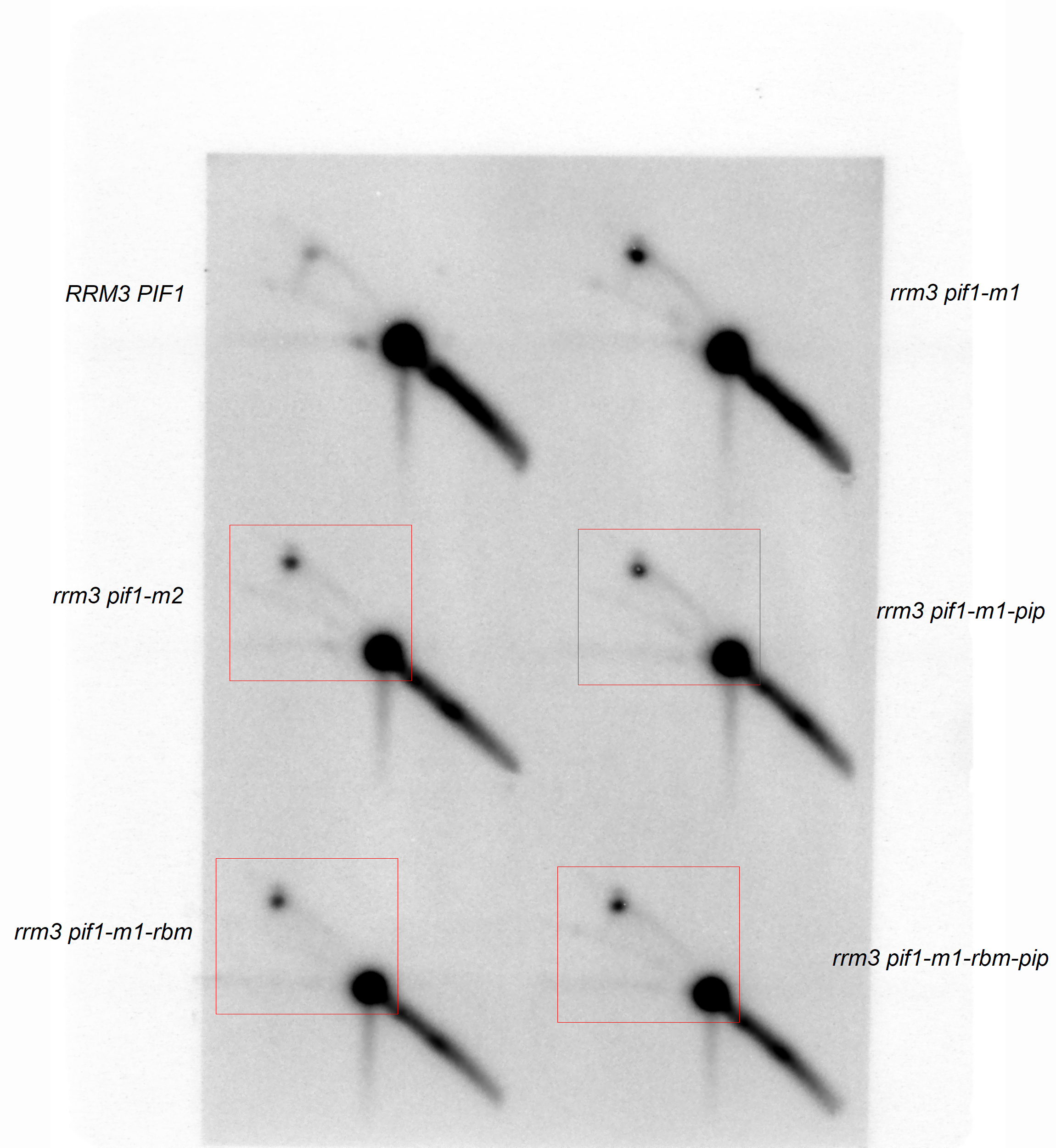

Supplement: Supplementary file 7 — Source Data Fig. 3 [file 44319_2024_114_MOESM7_ESM.zip › Panel B/tRNA southern part 2.tif]

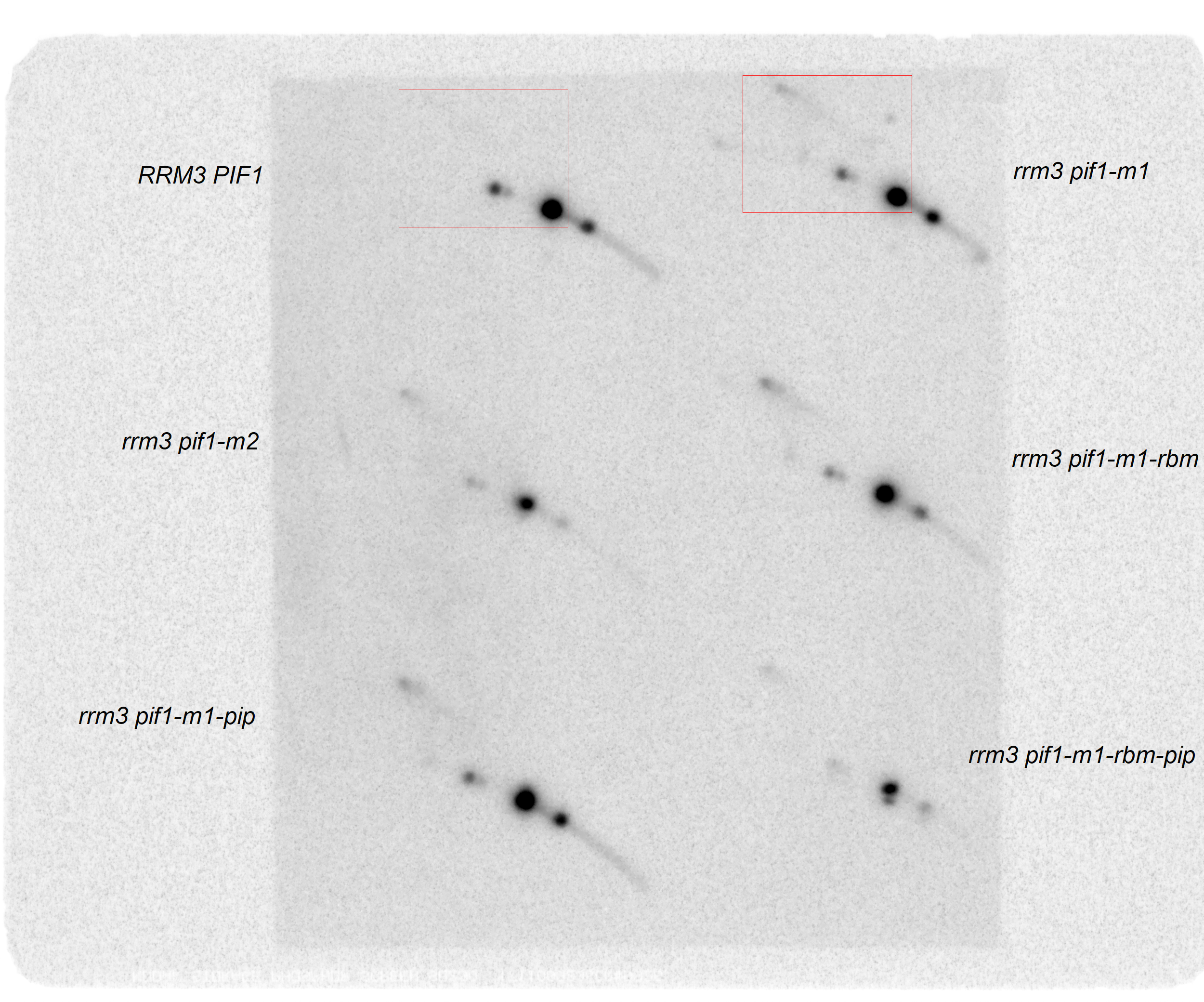

Supplement: Supplementary file 7 — Source Data Fig. 3 [file 44319_2024_114_MOESM7_ESM.zip › Panel D/URA3 southern part 1.tif]

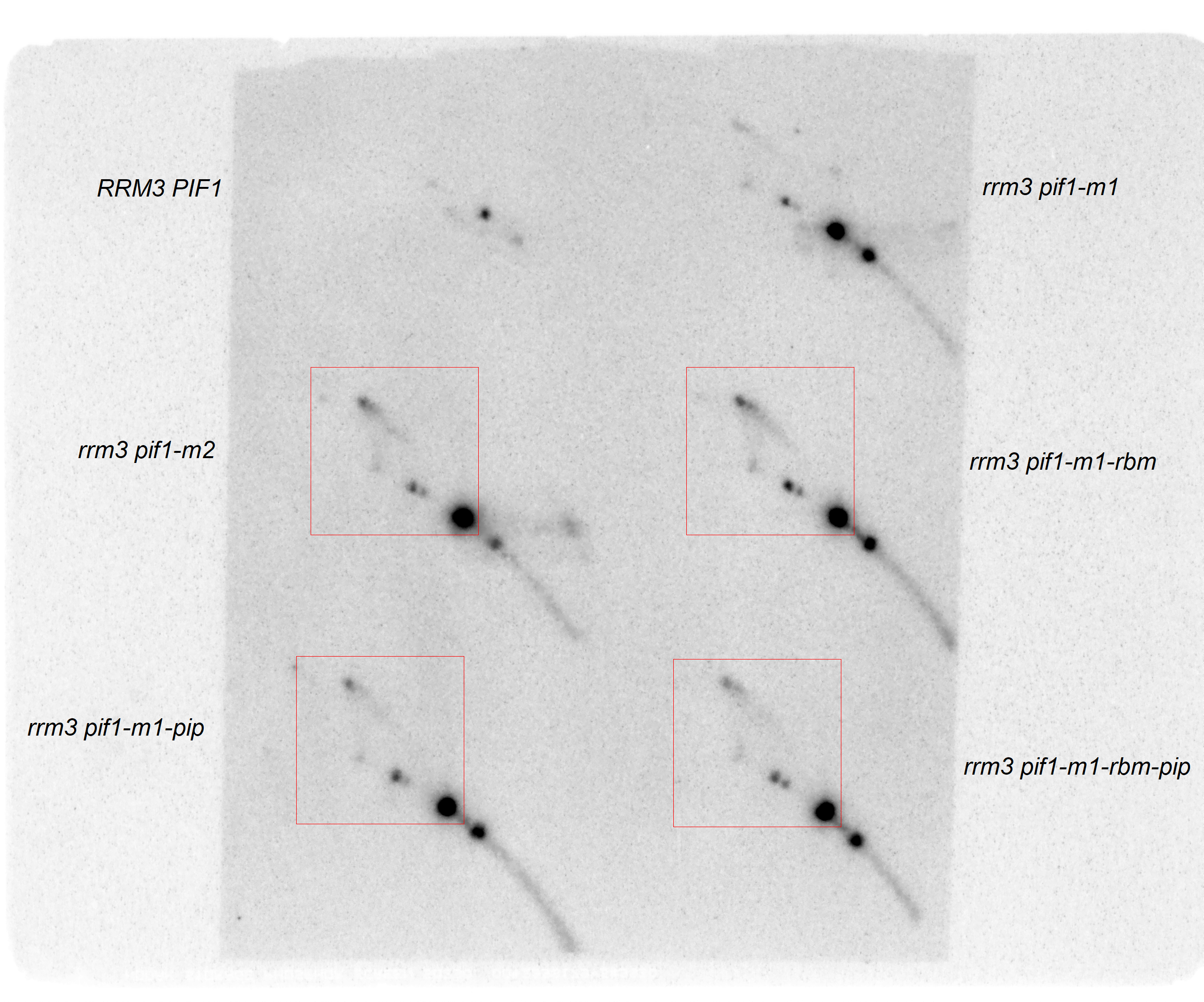

Supplement: Supplementary file 7 — Source Data Fig. 3 [file 44319_2024_114_MOESM7_ESM.zip › Panel D/URA3 southern part 2.tif]
