# Supplementary material for: The functional significance of the RPA- and PCNA-dependent recruitment of Pif1 to DNA
Source: EMBO Rep. 2024 Mar 13;25(4):10. doi: 10.1038/s44319-024-00114-9 (PMC11014909; doi:10.1038/s44319-024-00114-9)
Supplement: Supplementary file 9 — Expanded View Figures [file 44319_2024_114_MOESM9_ESM.pdf]

Expanded View Figures

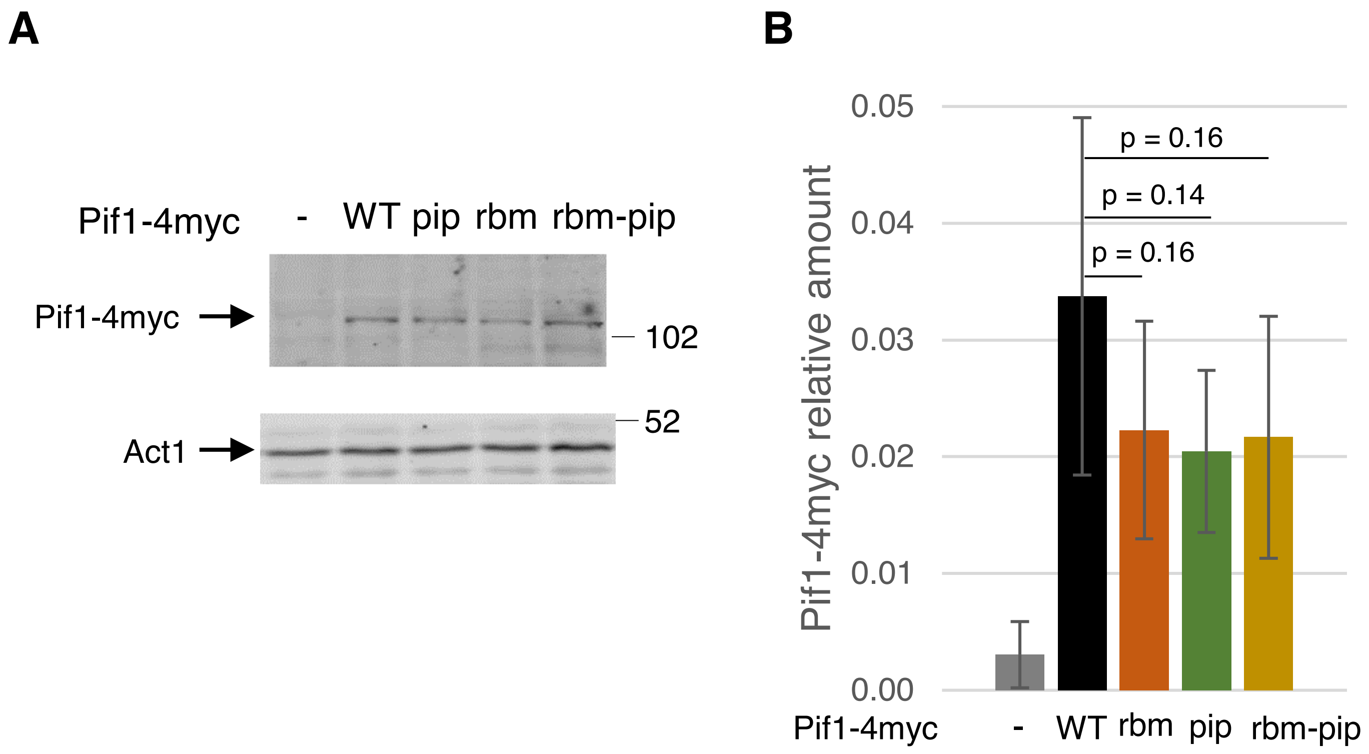

Figure EV1. Mutations in RBM and PIP motifs of Pif1 do not affect the protein levels.

(**A**) Western blot analysis of Pif1 and Act1 from the total cell protein samples prepared from yeast log-phase liquid cultures (a representative image). The numbers on the right indicate the molecular weight (in kDa) of the size marker proteins run alongside the experimental samples. (**B**) Quantification of the Pif1 steady state levels from the experiments in (**A**) (normalised to Act1). Average values +/− SD are plotted ($n \geq 3$ biological replicates). Statistical significance was calculated by paired *t* test. Source data are available online for this figure.

 © The Author(s)

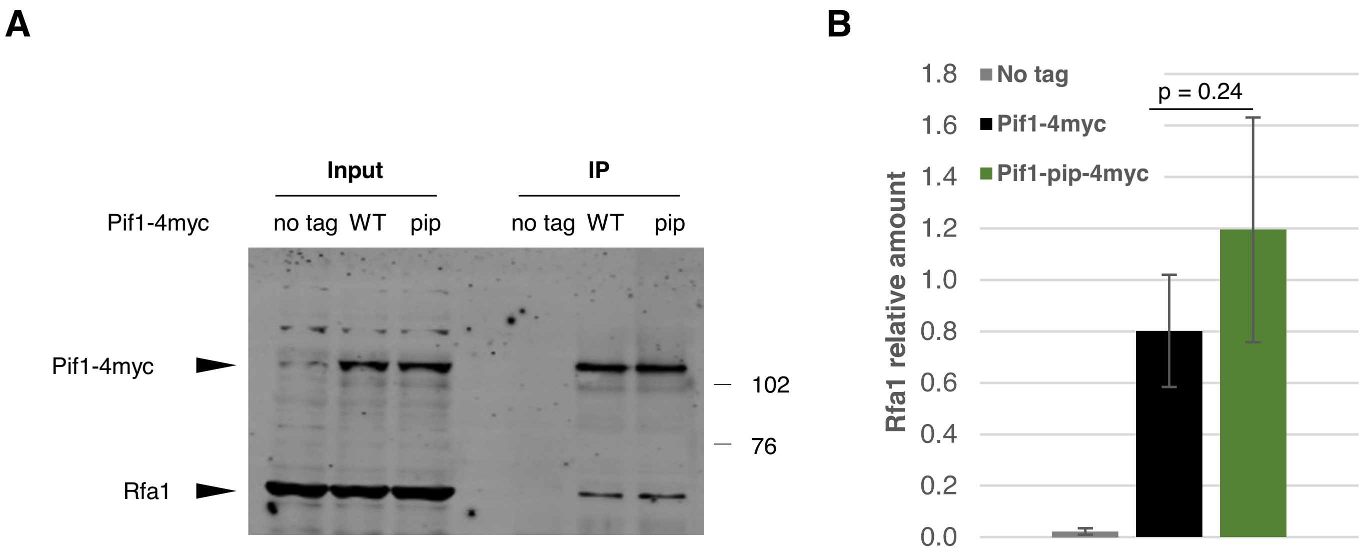

Figure EV2. The PIP motif of Pif1 is not required for the interaction between Pif1 and RPA.

(**A**) Proteins immunoprecipitated using anti-myc (9E10) antibodies were analysed by western blotting. Rfa1 was detected using anti-RPA (*S. cerevisiae*) antibody, Pif1-4myc and Pif1-pip-4myc were detected by anti-myc antibody. The numbers on the right indicate the molecular weight of the size marker proteins (in kDa) run alongside the experimental samples. (**B**) Quantification of the experiments in (**A**). Rfa1 signal was normalised to the corresponding input and to the relative myc signal. Average values +/− SD are plotted ($n = 3$ biological replicates). Statistical significance was calculated by paired t test. Source data are available online for this figure.

© The Author(s)

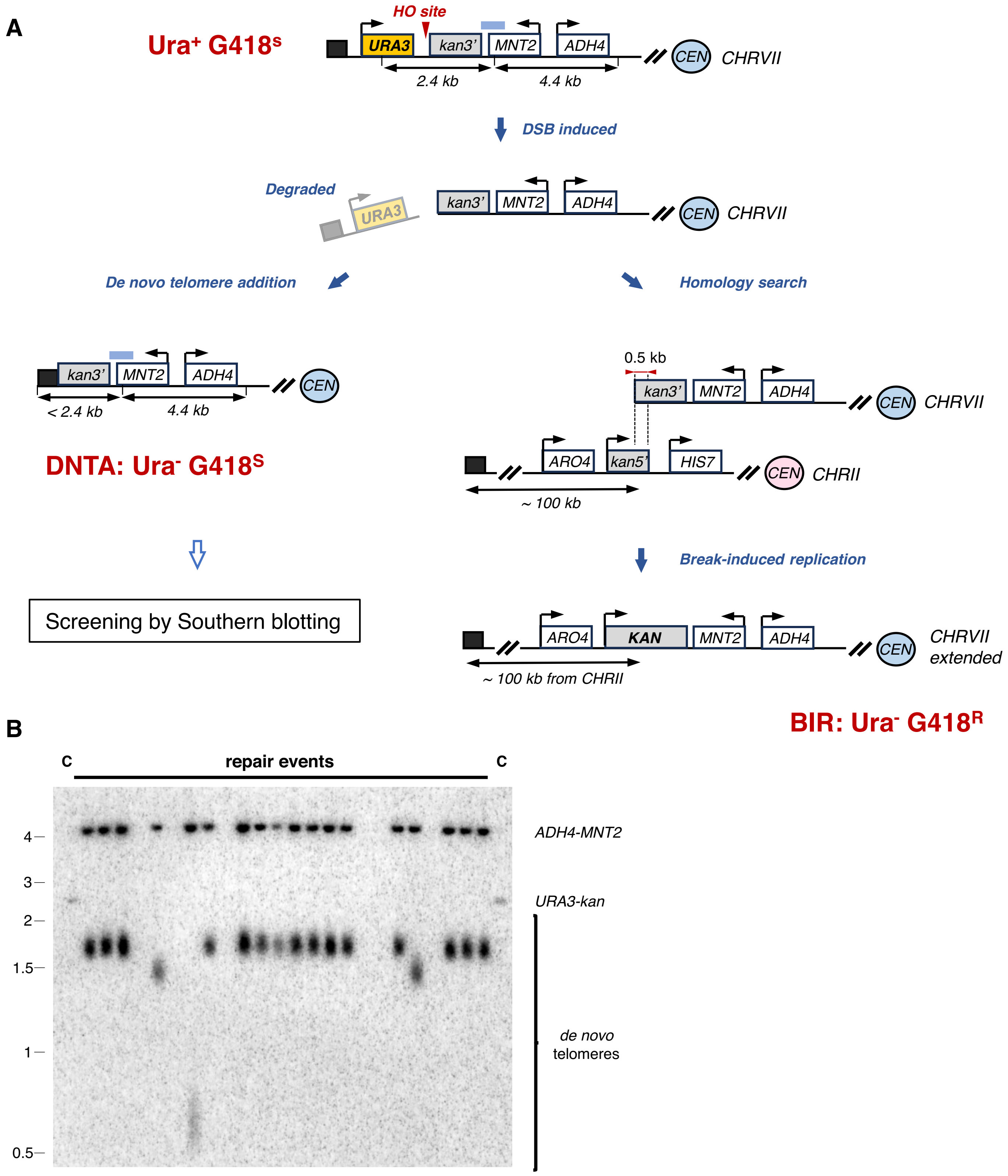
A
Ura+ G418s
HO site
URA3
kan3'
MNT2
ADH4
CEN
CHRVII
2.4 kb
4.4 kb
DSB induced
Degraded
URA3
De novo telomere addition
Homology search
< 2.4 kb
DNTA: Ura- G418S
0.5 kb
ARO4
kan5'
HIS7
CHRII
~ 100 kb
Break-induced replication
Screening by Southern blotting
KAN
CHRVII extended
~ 100 kb from CHRII
BIR: Ura- G418R
B
C
repair events
C
4
3
2
1.5
1
0.5
ADH4-MNT2
URA3-kan
de novo telomeres

 © The Author(s)

Figure EV3. Analyses of DNTA and BIR.

(**A**) Schematic of the genetic assay used to analyse the frequency of DSB repair by DNTA and BIR. The DSBs are generated by galactose-inducible expression of the HO-endonuclease in the strains with the HO-recognition site located at *MNT2 (CHRVIIL)*. The modified sub-telomere prior to the DSB induction contains the 3′ end of the *KAN* marker (confers a resistance to the drug G418), followed by the HO cleavage site, *URA3* (the endogenous *URA3* is mutated) and a telomere (black rectangle). Prior to the DSB induction, the cells are Ura^+ $G418^S$. After the *HO* induction by galactose, the *URA3-telomere* fragment is cleaved off and degraded by the break resection nucleases. The *kan-MNT2* DNA end can be healed by telomerase (the scenario on the left) generating Ura^- $G418^S$ cells, which are then to be screened by Southern blotting (**B**) to detect the actual *de novo* telomeres. For this, the genomic DNA is digested with EcoRV, resolved on an agarose gel and transferred onto a membrane. The DNA is probed with a *MNT2* probe spanning the EcoRV site located within *MNT2*. The lengths of the relevant EcoRV restriction fragments are shown by double arrowheads and the position of the probe on the chromosome by a blue rectangle. Alternatively, the *kan-MNT2* end can be repaired by BIR (the scenario on the right) involving a 0.5 kb *KAN* homology provided by the overlapping sequences between two incomplete versions of *KAN* indicated by the dashed lines. The completion of BIR leads to a reconstitution of the full-length *KAN* and an extension of the *CHRVIIL* arm by ~100 kb, due to copying the DNA sequence of *CHRII* from the point of the break invasion to the telomere. This generates Ura^- $G418^R$ colonies. (**B**) A representative image of a Southern blot used to screen for Ura^- $G418^S$ colonies for DNTA events. Lanes marked with C contain samples from the cells before the break induction. The numbers on the left indicate the molecular weight (in kb) of the DNA size marker fragments run alongside the experimental samples. Source data are available online for this figure.

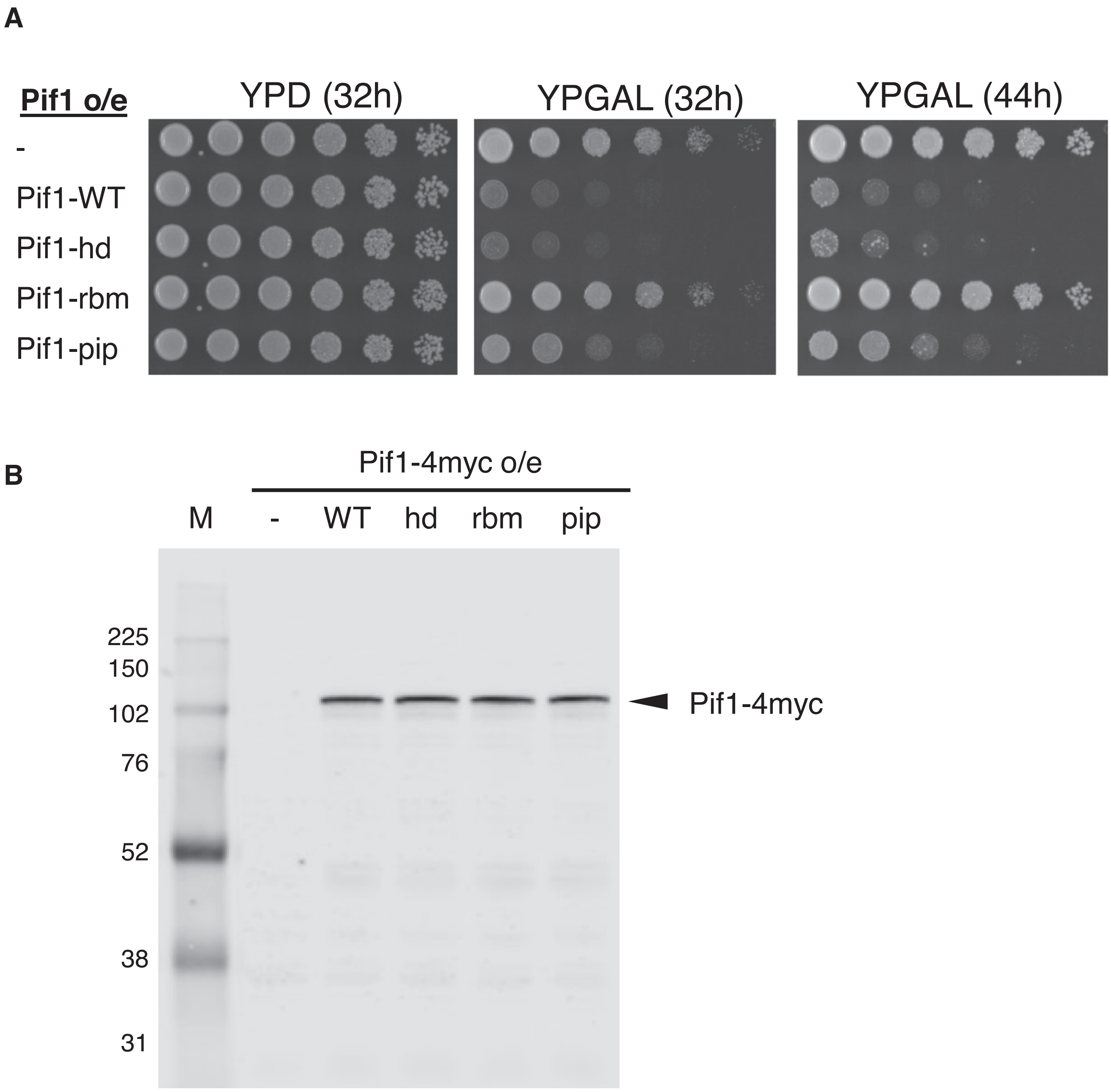

Figure EV4. Overexpression of Pif1 is toxic due to its ability to interact with RPA and PCNA. (**A**) Fivefold serial dilutions of the freshly grown isogenic strains were spotted on YPD and YPGAL plates and incubated at 30 °C for the duration shown above the images. (**B**) Comparative analysis of the relative Pif1-4myc protein levels in YPGAL for the strains analysed in panel A. Galactose was added to the log-phase cultures grown in YPRAF and after 3 h of additional culturing cells were harvested and the total cell lysates were analysed by western blotting. Pif1-hd is a helicase-dead Pif1 derivative containing the previously characterised K264A substitution.

 © The Author(s)
